# Decolonisation of the nursing education curriculum in Gauteng province, South Africa: A concept analysis

**DOI:** 10.4102/hsag.v28i0.2449

**Published:** 2023-12-08

**Authors:** Agnes Makhene

**Affiliations:** 1 Department of Nursing, Faculty of Health Sciences, University of Johannesburg, Johannesburg, South Africa

**Keywords:** decolonisation, nursing education, curriculum, concept analysis, curriculum

## Abstract

**Background:**

Decolonisation of the nursing education curriculum has become more important than ever. The nursing profession has been colonised since its founding era by Florence Nightingale. Victorian curriculum has been taught over decades in nursing. There is a knowledge gap of what decolonisation means in the profession.

**Aim:**

The purpose of this article is to describe the concept analysis process that was followed to clarify the concept of ‘decolonisation’ of the curriculum, provide conceptual meaning in nursing education, and formulate a theoretical definition.

**Setting:**

Nursing education practice in Gauteng.

**Methods:**

Definitions, nature, characteristics, and uses of decolonisation were sought and the researchers explored 52 publications, which included dictionaries, encyclopaedias, thesauri, conference articles, research reports, journal articles and subject-related literature across multiple disciplines, to critically analyse the concept ‘decolonisation’. A 10-year period from 2012 to 2022 was used to search several databases.

**Results:**

Defining attributes that included antecedents, process and consequences of decolonisation emerged. The antecedents were awareness, identification of colonial knowledge and thought, colonial attitudes, colonialism, racism, exclusion, denial of colonial systems and curriculum and colonial legacies in nursing education. Events after decolonisation are called consequences.

**Conclusion:**

The formulated theoretical definition of ‘decolonisation’ will be operationalised as further research takes place to come up with a conceptual framework for a decolonised socially just nursing curriculum.

**Contribution:**

This study aimed to contribute towards the understanding of what decolonisation means within nursing education and lead to development of models, theories, and strategies on how decolonisation of the nursing curriculum can be undertaken.

## Introduction

Decolonisation of the nursing education curriculum has become more important than ever. The nursing profession has been colonised since its founding era by Florence Nightingale. This Victorian curriculum has been carried out through decades of the existence of the nursing profession. There is a knowledge gap of how a decolonised nursing education curriculum should look like. Decolonisation is a broad concept that is applied differently in many contexts, including the education setting. A decolonised education means that the curriculum needs to be transformed to reflect the lived experiences of African people, including recognition of their scholarly work, which is often on the periphery or taught as additional modules. It was argued that decolonised education means recentring the work of these scholars in the curriculum. Nursing education curriculum as applied in South Africa has predominately been developed by European scholars. Colonisation of African countries by European settlers also resulted in the colonisation of education.

Historically, nursing education in South Africa was established by Henrietta Stockdale in Kimberley in the 1880s (Dolamo & Olubiyi 2013). According to McGibbon et al. (2014), colonial historical periods are not considered influential or relevant in contemporary nursing and are hence not valued in nursing discourse. In 2015 and 2016, the ‘#Fees must fall’ student protest made its debut in South Africa. Nursing students were included in these student protests because all university students demanded that the curriculum, which is founded on Eurocentric epistemology, philosophies, ideas, and beliefs, be decolonised. Le Grange (2018) argues that while first-generation colonialism focused on enslaving the colonised people’s physical locations and bodies, second-generation colonialism focused on enslaving people’s minds through fields such as science, economics, law, and health.

Neo-colonialism relates to the achievement of technical independence of a country that is still under the influence of the ex-colonial or newly developed superpowers. Put simply, decolonisation is the ‘undoing of colonization’ (Le Grange 2018). The need to decolonise nursing education curriculum has become more important, especially in the 21st century and the fourth industrial revolution as evidenced by a decrease in resources during the advent of the COVID-19 pandemic.

## Research problem

The nursing education curriculum has been based on colonial frameworks, for example, frameworks by nursing theorists such as Watson and Peplau dominate the field. The researcher observed that there is no conceptual framework for a decolonised nursing education curriculum in South Africa. This necessitated the analysis of the concept ‘decolonisation’ within nursing education, which will culminate in a theoretical definition of this concept.

### Research question

What is the conceptual meaning of the concept ‘decolonisation’ within the context of nursing education?

### Research objectives

The objectives of this article include:

- to clarify the conceptual meaning of the concept ‘decolonisation’ within the context of nursing education;
- to provide a conceptual meaning in nursing education;
- to formulate a theoretical definition of decolonisation in nursing education.

### Research method

The concept analysis method developed by Walker and Avant (2019) was used to analyse decolonisation. This approach, which was deemed best by Tofthagen and Fagerstrm (in Thompson 2018), was chosen as it is the most straightforward, thorough, and methodical approach, making it simpler to comprehend and employ with concepts that are still developing (Yazdani & Shokooh 2018). According to Walker and Avant (2019), the eight steps of this strategy are not required to be sequential (Thompson 2018). The eight steps are: (1) selecting a concept, (2) determining the aims or purpose of analysis, (3) identifying all uses of the concept, (4) determining the defining attributes of the concept, (5) constructing a model case, (6) constructing borderline, contrary, invented, and illegitimate cases, (7) identifying antecedents, (8) defining empirical referents.

#### Step 1: Selecting a concept

The importance of any concept depends on the range of different factors in the field and its outside boundaries over time. The core of concept analysis is selecting the defining attributes (Walker & Avant 2019). Therefore, a concept for which there is no clear definition should be analysed to clarify it. Decolonisation has been given different meanings in different settings, making it ambiguous. Thus, this necessitated the selection of this concept for analysis by the researcher (Sharifi, Adib-Hajbaghery & Najafi 2019). Students in higher education institutions in South Africa have been calling for decolonisation of the curriculum, including nursing education. However, there is no clear definition of the concept of decolonisation in nursing education to assist potential researchers with the development of a framework or model of decolonising the nursing curriculum.

#### Step 2: Determining the purposes of analysis

The first purpose of the analysis was to clarify the conceptual meaning of decolonisation in nursing education by identifying the defining attributes of the concept. The second purpose was to formulate a theoretical definition of decolonisation, a process that will enable formulation of an operational definition to assist potential researchers to develop models or conceptual frameworks that can be used to develop decolonised curricula or formulate strategies that can be used in decolonisation of the curriculum using the identified attributes (Walker & Avant 2019).

#### Step 3: Identifying all uses of the concept (sample and sampling method)

The researcher studied dictionaries, encyclopaedias, thesauri, conference articles, research reports, journal articles, and subject-related literature from a variety of fields to critically assess decolonisation (Walker & Avant 2019). The researcher was only interested in the definitions, nature, characteristics, and applications of decolonisation that were available in the literature. This body of literature provided the researcher with rich data, a deeper understanding of the meaning, and crucial details required for careful study (Denzin & Lincoln 2018).

According to Walker and Avant (2019), significant literature from a range of disciplines and fields of study, including education, psychology, sociology, engineering, medicine, and nursing, should be included to critically analyse the concept. The Cumulative Index of Nursing and Allied Health Literature (CINAHL), MEDLINE, Education Resource Information Center (ERIC), Google Scholar, PsycInfo, Index to Dissertations and Theses, and a complex search technique were used to accomplish this. The researcher utilised search phrases such as ‘colonise’, ‘decolonise’, and ‘decolonisation of the curriculum’ to find literature from both primary and secondary sources. The scope of the search was expanded to encompass all relevant meanings of the concept and was not limited to nursing.

A total of 670 publications were identified from 5119 publications that were restricted to academic journals, conference articles, full-text articles with references, corrections for double entries, and English language. Data saturation was realised at article 52 whereby definitions, nature, characteristics, and applications of decolonisation were described (Polit & Beck 2021). Out of the 52 sources, 14 were dictionaries, thesauri, and encyclopaedias definitions that were selected for concept analysis.

**FIGURE 1:**
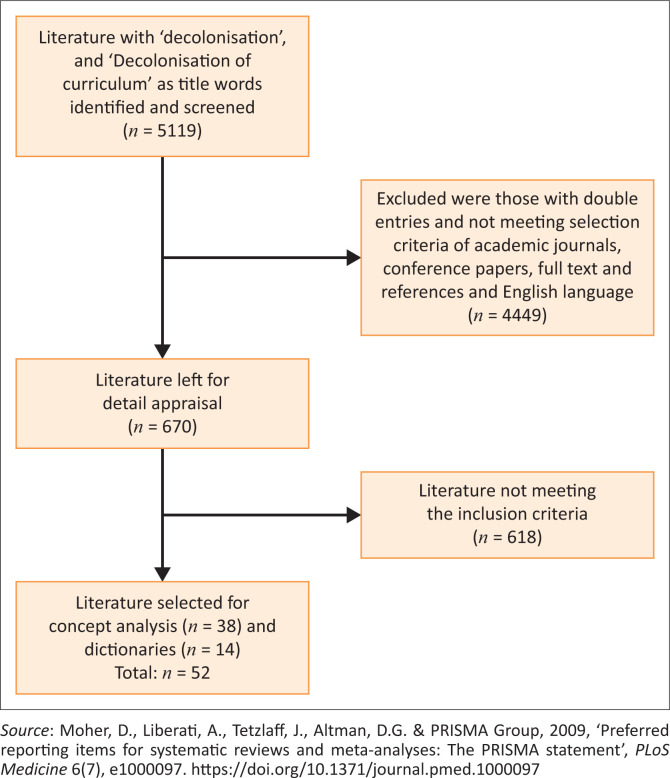
The Preferred Reporting Items for Systematic Reviews and Meta-Analysis (PRISMA).

To ensure that relevant information and substantial early work related to decolonisation was included, the inclusion criteria covered literature from the year 2012 to 2022, a 10-year range. This was done to cover varied research on decolonising the curriculum. The literature had to exclusively explore the definitions, nature, characteristics, and applications of decolonisation of the curriculum. It also had to be fully text-searchable and published in English. Literature that did not contain information on the definitions, characteristics, or applications of decolonisation as well as non-English publications, copies, and irrelevant works that did not fall under any extra limitations were deleted. The exclusion criteria were double entries, non-academic journals and not written in the English language. The inclusion and exclusion criteria are shown in the PRISMA flow chart in Figure 1.

#### Step 4: Determining the defining attributes

The suitability and accuracy of the information from the literature had an impact on the researcher’s sound reasoning (Thompson 2018) used to identify the defining attributes. The concept analysis was undertaken to identify a group of attributes that have a strong relationship with the concept and allowed the researcher to obtain deeper insight about the concept (Walker & Avant 2019). The researcher immersed herself in the definitions, nature, characteristics, and uses of decolonisation in a repetitive and iterative process. A three columned table was used to analyse data from concept definition in column one to reduction, using concept synthesis in column two and final reduction using process of concept derivation in the third column. Content analysis was employed to identify similar and contrary patterns to formulate meaningful statements talking to the nature, characteristics, and attributes of ‘decolonisation’. The researcher began with sorting the codes by their similarities and differences and abstracting them into sub-categories and eventually categories. This was performed by dividing the original text into meaning units and condensing and coding those units (Lindgren, Lundman & Graneheim 2020).

#### Step 5: Identifying antecedents (precursor) and consequences

Antecedents are the prerequisites that must be met for decolonisation of the curriculum to take place (Walker & Avant 2019). These were described as racism, fundamental change, awareness of colonial knowledge and ideas, colonial structures and curriculum materials, and the overall impact of colonialism on nursing education institutions. The events that follow decolonisation are referred to as consequences (Walker & Avant 2019). Decolonising the nursing curriculum will lead to the development of learning environments that do not tolerate stereotypes and prejudice.

#### Step 6: Defining empirical referents

The existence of the concept is determined by empirical referents, which are classifications or categories. ‘Empirical referents are classes or categories of actual phenomena that by their existence or presence demonstrate the occurrence of the concept itself’ (Walker & Avant 2019). These are how one can measure the defining characteristics or attributes. They include new forms of knowledge, social justice and redress in nursing education, inclusive educational spaces and inclusion of indigenous knowledge in the curriculum.

#### Step 7: Identifying a model case

A model case was identified using a curriculum result, and it is shown with a different colour font and in bold. The model case below illustrates the concept analysis’ results, which represented the researcher’s best understanding of the notion of decolonisation in nursing education. The objective is to outline the use of the defining attributes of decolonisation of the curriculum that emerged during concept analysis (Walker & Avant 2019).

**Model case:** The purpose of this curriculum is to change (*fundamentally transform, reconstruct*) Eurocentric nursing knowledge perspectives (*colonial knowledge and thought, hegemonies, colonial attitudes, colonialism, racism, exclusion and denial colonial systems and content in curriculum and colonial legacies on nursing education systems*) and provide a conducive teaching and learning environment that includes indigenous knowledge nursing systems (*Africanising knowledge, creating new forms of knowledge that includes different indigenous knowledge systems using paradigms of indigenous knowledge and worldviews that inform self-determination practices, teachings and experiences*) to produce a nurse graduate that is indigenously aware and conscious to deliver all-inclusive and socially just nursing care (*gain foundational knowledge of indigenous knowledge systems and practices that calls on individual transformation and reconciliation within one’s self and beyond the scope of nursing education and socially just practice*).

#### Step 8: Identifying borderline, related and contrary cases (additional cases)

The model and the opposing case gave enough understanding to clearly separate the concept that there was no need to develop borderline and related situations. The words ‘important’, ‘central’, ‘major’, ‘imperialism’, ‘intolerance’, ‘irrefutable’, ‘ethnic’, and ‘alteration’ would have been present in a borderline case. The words ‘true’, ‘real’, and ‘original’ would have been used in a similar situation. There was no need to come up with a contrary case as the concept ‘decolonisation’ does not involve experimentation or laboratory studies (Walker & Avant 2019).

Table 1 display the articles that were selected and research methods that were assessed to ensure the rigor of the findings.

**TABLE 1:** Articles analysed and synthesised.

Author and year	Purpose	Design	Sample	Rigour and trustworthiness
Ahmed-Landeryou (2023)	To reflects on the development of an evidence informed ‘decolonising the curriculum wheel – a reflection framework’, as a method to support and guide engagement and continuous evaluation of decolonising curricula activities across a higher education institution, to potentially improve belonging for black and minoritised students.	Scoping review	32	Integrity of scoping review was ensured by adherence to reporting standards supporting well-informed decision-making.
Ali et al. (2021)	To highlight the impact of racial disparities on the educational experiences of black and minority ethnic students in healthcare education.	Qualitative ethnography	49	Lincoln & Guba’s credibility, transferability, dependability, confirmability and authenticity (Polit & Beck 2021).
Arday, Zoe Belluigi and Thomas (2021)	To explore the impact of a dominant Eurocentric curriculum within higher education and the influence of this upon navigating factors such as BAME attainment, engagement and belonging within the academy.	Qualitative	20	Establishing positionality and proximity to the research was essential in an attempt to acknowledge and reduce researcher bias from an ethical perspective. There was some acknowledgement that the researchers were closely associated with racialised discourses. Therefore, some organic bias was inevitably inherent, although all protocols were administered to ensure objectivity was maintained and any potential biases were minimised throughout the study.
Bell (2020)	To review racism, anti-racism, whiteness, nursing education and nurse educators and analyse for the development of race consciousness and application of anti-racist pedagogy.	Qualitative	50	Credibility, transferability, dependability, confirmability and authenticity.
Burnett et al. (2020)	To explore indigenous people’s beliefs about vaccination to complicate notions of rurality in order to illuminate the ways in which space and settler colonialism both shape and limit choices around health care access.	Qualitative	86	The study used MAXQDA qualitative software analysis to code and assist with analysing transcripts. The software allowed for organisation and management of the data thematically as the study had a large number of participants (over 70). The software also enabled comparing and ensuring that the excerpts the authors highlighted were consistent with what participants shared with them across focus groups.
Curtis et al. (2019)	To inform the Medical Council of New Zealand, in reviewing and updating its approach to cultural competency requirements Curtis et al. *International Journal for Equity in Health* (2019) 18:174 Page 2 of 17 for medical practitioners in New Zealand Aotearoa.	Literature review	59 articles	This review and analysis has been conducted from an indigenous research positioning that draws from Kaupapa Māori theoretical and research approaches. Therefore, the positioning used to undertake this work aligns to effective Kaupapa Māori research practice that has been described by Curtis (2016) as: transformative; beneficial to Māori; under Māori control; informed by Māori knowledge; aligned with a structural determinants approach to critique issues of power, privilege and racism and promote social justice; non-victim-blaming and rejecting of cultural deficit theories; emancipatory and supportive of decolonisation; accepting of diverse Māori realities and rejecting of cultural essentialism; an exemplar of excellence; and free to dream.
Dolamo and Olubiyi (2013)	To assist modern states in Africa to learn from the past in order to make good decisions for the present as they build a future that will make nursing education a key and crucial partner in the management of health services, including attainment of some of the millennium development goals.	Literature review- Historical	Document analysis	None
Drummond, Cox and Best (2016)	To continue the critical discourse regarding the decolonisation of nursing education and specifically to assert the obvious evolution for Australian nursing education given the social inequities that stem from Australia’s (comparably more recent) colonial history.	Literature review- conference presentation	None	Not mentioned
Du Plessis (2021)	To explore the tensions, challenges and complexities inherent in the heads of department’s leadership practices.	Qualitative- Utilising a decolonised education and a social justice leadership discourse framework,	Five university HOD’s – purposeful sampling	For data verification and authentication purposes, each leadership challenge had to be cited by at least three interview participants in order to be recognised for the study.
Eichbaum et al. (2021)	Analysis of the intersections between colonialism, medicine, and global health education and explore the lingering impact of colonialist legacies on current global health programmes and partnerships.	Literature review	43	A thorough, systematic, and iterative review of the literature, and the use of the results to establish a clear analysis.
English (2021)	To explore efforts at personal reconciliation as a settler nurse educator. As part of these efforts, the author includes an analysis of their experience within the context of the nursing profession’s necessity to meet relevant calls to action outlined within the Truth and Reconciliation Commission’s report.	Reflective narrative	Not described	Not described
Fontenot and McMurray (2020)	To examine the current recruiting practices for prelicensure nursing programmes within the United States and using existing research, inspire various methods to decolonise the recruiting and subsequent application process to expand the diversity of applicants.	Literature review	31	A thorough, systematic, and iterative review of the literature to obtain accurate findings.
Goodman et al. (2017)	To explore the healthcare experiences of Aboriginal peoples who use illicit drugs and/or illicit alcohol (APWUID/A) living in Vancouver’s inner city.	Indigenous research methodology	30	An iterative process ensured the presentation of the findings in this article is an accurate portrayal of community members’ healthcare experiences, analysis and interpretation of the data.
Heleta (2016)	To trace the roots of Eurocentrism and epistemic violence at universities.	Reflective narrative	Not described	Not described
Kerr (2014)	To explore the dynamics of contemporary colonialism in the Canadian settler nation-state in the context of programs of teacher education.	Reflective case study narrative	Not described	Not described
Khuzwayo (2021)	To explore views, opinions, and ideas of pre-service teachers that could influence the development of an alternative paradigm for empowering teachers with the competence to promote a balanced worldview in teaching and learning in schools.	Qualitative- case study research design	340 students 12 in a group – 34 groups	Collaboration, engagement, and participation were encouraged and monitored during the process to ensure that all students received the opportunity to express their views.
Kimani (2023)	To critically discuss the intersections between racism and colonialism as social determinants of health and explore how these discriminatory ideologies shape nursing inquiry.	A literature review of pertinent discourse on racism and colonialism in nursing Discursive paper using pertinent nursing literature.	Articles from 2000 to 2022 Sample size not specified	An iterative process ensured accurate presentation of study methods and findings.
Iheduru-Anderson (2020)	To explore how the perception of racism or racial bias affects the motivation of black or African American nurses (BAANs) in the United States (US) to seek and apply for nursing leadership and faculty positions, and to characterise the racism-related barriers that BAANs perceive that prevent them from moving forward with their careers in academia and nursing leadership.	Qualitative ethnographic	30	Credibility, transferability, dependability, confirmability and authenticity.
Le Grange (2018)	To clarify what is meant by the internationalising, indigenising, decolonising, and Africanising of the curriculum. To discuss the ways in which the concepts are disparate and explore the conceptual connections between and among them.	Literature review	36	Depth, objectivity, currency, authority, and purpose.
Meda (2020)	To explore students’ perspectives about decolonising the curriculum.	Qualitative case study	10	Credibility, transferability, dependability, confirmability and authenticity.
McCleland (2011)	To define cultural safety and the concern that indigenous students put aside their culture when they commit to study within an education steeped in Westernised thinking. Discusses the decolonisation of research and the part that non-indigenous researchers play within an indigenous research project.	Qualitative literature review	Metaphorical whanau groups – sample size not specified	Depth, objectivity, currency, authority, and purpose.
McGibbon et al. (2014)	To underscore the urgent need to further articulate postcolonial theory in nursing and to contribute to nursing knowledge about paths to work towards decolonising the profession.	Literature review	Not described	Depth, objectivity, currency, authority, and purpose.
McGregor and Park et al. (2019)	To argue for the deconstruction rather than the decolonisation of the neocolonial curriculum.	Case study design	Two case studies	Depth, objectivity, currency, authority, and purpose.
Mignolo and Walsh (2018)	To describe thinking decolonially as Geopolitics of Sensing and Knowing: concerned with global equality and economic justice, asserting that Western democracy and socialism are not the only two models to orient thinking and doing.	Qualitative ethnographic literature review	15	Depth, objectivity, currency, authority, and purpose.
Morreira et al. (2020)	To examine the relationship between decolonisation as a theoretical concept, and the practices of decoloniality unfolding in pedagogical practice.	Theory generating	Not described	Depth, objectivity, currency, authority, and purpose.
Nazar et al. (2015)	To highlight the impact of three models of cultural diversity education and identified that while the cultural competence approach reinforces notions of difference consistent with neo-colonialism, the cultural humility model aligns with patient-centred care.	Qualitative	15	Credibility, transferability, dependability, confirmability and authenticity.
Ngunyulu, Mulaudzi and Peu (2020)	To explore and describe the perspectives of nursing students regarding incorporating ATIK into the curriculum.	A participatory transformative approach	39	Credibility, transferability, dependability, confirmability and authenticity.
Rowe, Baldry and Earles (2015)	To analyse the relationship between indigenous and critical approaches, demonstrate how dominant knowledge and power relationships can be transformed by prioritising the unique ontological, epistemological, and axiological positioning of indigenous approaches.	Qualitative	Not described	Credibility, transferability, dependability, confirmability and authenticity.
Sharifi et al. (2019)	To analyse the concept of cultural competence in nursing.	Concept analysis	43	Depth, objectivity, currency, authority, and purpose.
Seyama (2019)	To undertake conceptual analysis of decolonising the curriculum.	Qualitative	35	Depth, objectivity, currency, authority, and purpose.
Tuck and Yang (2012)	To explore what is unsettling about decolonisation. Analyse varied settlers’ moves to innocence in order to foster ‘an ethics of incommensurability’, acknowledging what is different and sovereign for decolonisation projects as related to social justice projects based on human and citizen’s rights.	Qualitative		Depth, objectivity, currency, authority, and purpose.
Thurman et al. (2019)	To identify how and to what extent peer-reviewed nursing literature and professional nursing organisations have explicitly addressed institutionalised racism.	Integrative literature review	29	Depth, objectivity, currency, authority, and purpose.
Waite and Nardi (2019)	To explore the nurse leader’s role in understanding the impact of American colonialism – specifically racism, a product of colonialism – as a key determinant in shaping the education of nursing students and its influence on practising nurses.	Qualitative literature review- historical document analysis	25	Depth, objectivity, currency, authority, and purpose.
Wong, Gishen, and Lokugamage (2021)	To describe the pioneering efforts to decolonise the undergraduate medical curriculum at UCL Medical School (UCLMS), London.	Case study	Students, faculty- sample size not specified	Depth, objectivity, currency, authority, and purpose.
Zembylas (2023)	To use decolonial thinking, as applied in the field of AI, to explore the ethical and pedagogical implications for higher education teaching and learning.	Qualitative	56	Depth, objectivity, currency, authority, and purpose.

*Source:* Adapted from Balcombe, L., Miller, C. & McGuiness, W., 2017, ‘Approaches to the application and removal of compression therapy: A literature review’, *British Journal of Community Nursing* 22, S6–S14. https://doi.org.proxy1.cl.msu.edu/10.12968/bjcn.2017.22.Sup10.S6

AI, artificial intelligence; BAME, Black, Asian, and minority ethnic; ATIK, African traditional indigenous knowledge; HOD, Head of department.

## Findings

The results of the concept analysis are described according to the three categories of decolonisation in nursing education: antecedents, process, and outcome. Moreover, a theoretical definition of decolonisation was formulated.

### Antecedents

Recognising the biases in the nursing curriculum that result from medical education and training is the first step in decolonising nursing education. One cannot begin to conceive what a postcolonial or decolonial reality may look like in a nursing curriculum until the public sector acknowledge that institutionalised forms of coloniality still exist in the society today (Wong et al. 2021). The first view of decolonisation calls for a paradigm shift to advance social justice, social and cultural identities, and to correct Eurocentric superiority and imperial attitudes (Khuzwayo 2021). There is an enormous number of Euro-American sources of information in nursing and health sciences. The textbooks, Internet resources and professional journals stem mostly from sources outside Africa. Du Plessis (2021) refers to ‘western learning’ as a place where students become entrapped in one worldview in that a mono-cultural monopoly is being presented. Prescribed textbooks given to African students for learning still populate a greater number of international authors, which creates a great challenge for students to be able to contextualise the content to the real needs of the country. The call is upon South African scholars to embark on producing academic resources for Africa that address the African community so that the percentage of international texts can be reduced. Transformative learning shifts the emphasis from the nurse educator to the student, with the educator creating the holistic learning experiences to empower the students. Learning can be defined here as ‘a change in behaviour (knowledge, attitudes and/or skills) that can be observed or measured and that occurs because of exposure to environmental stimuli’ (Bastable & Myers 2014). Sadly, nursing education has a long history of oppression, submission, and subordination, waiving off positive development for transformation. Studies also highlight that students express difficulties with creating safe spaces for dialogue (McGibbon et al. 2014).

The broad objectives of the present tradition of decolonial philosophy include combating the negative effects of colonialism, opposing Western Eurocentrism and the ways it oppresses colonially disempowered peoples, and exploring one’s relationships to the universality of Western thought (Mbembe 2016; Mignolo & Walsh 2018). Decolonial frameworks offer useful tools for examining how Western, colonial, and Eurocentric epistemological foundations influence the teaching and discussions on how people differ (Zembylas 2023). These foundations support the idea that there is one universal world made up of major and minor groups, with a ‘human’ who is supported by a colonial epistemological ‘Western Man.’

Decolonisation is the disruption or destruction of systems that support laws and practices that maintain white supremacy, promote discrimination, restrict diversity, and hinder equity. To remove whiteness in nursing programmes, it is necessary to eliminate practices and procedures that support white supremacy, such as those governing faculty recruiting, retention, and learner health and wellness (Fontenot & McMurray 2020). Current nursing education practices must be dissected to create fresh programmes and regulations. Decolonising nursing practice requires its practitioners to question the discipline’s biological modernist paradigm (Drummond et al. 2016). In addition to carrying out their clinical responsibilities, nurses should look for chances to question the presumptions and philosophical foundations of their profession.

In a paradigm shift, decentring the frame of reference from the lens of a dominant group to that of a post-colonial legacy who are at the intersections of and differentially vulnerable to forms of social oppression may help nurse educators to uncover the epistemological influences of the colonial legacy on their teaching practice. This principle of two-eyed seeing entails a bifocal consideration of indigenous and non-indigenous ways of knowing (not one in isolation) (Morreira et al. 2020). According to Burns (2015), focusing too heavily on the Western model of thought could undermine indigenous wisdom. Everyone concerned must participate in an ongoing process of collective ‘conscientisation’, learn to recognise one’s own beliefs and behaviours, whether they are indigenous people or not, and test whether they are influenced by colonial presumptions (Freire 2000). To give voice to alternative tales and constructions of reality, decolonising methodology seeks to deconstruct the institutional processes and narratives (Smith 2012). As a result, decolonising knowledge in universities entails a keen awareness of and opposition to colonial forms of knowledge, teaching approaches, and research paradigms. It is significant to highlight that, in comparison to earlier incarnations of the culturally oriented techniques indicated above, the cultural safety and cultural humility pedagogies use a more critical focus. Cultural safety encompasses a critical consciousness where healthcare professionals and healthcare organisations engage in ongoing self-reflection and self-awareness and hold themselves accountable for providing culturally safe care, as defined by the patient and their communities, and as measured through progress towards achieving health equity (Curtis et al. 2019).

The issue with these techniques is that they permit learners to advance through training without carefully examining their racial socialisation or their understanding of culture when explicit anti-racist teaching is abandoned in favour of a culturally oriented pedagogy. Cultural humility may inspire people to evaluate socio-political issues. The depiction of race as a social construct rather than a biological one, whether overtly stated or not, is a significant recurring error in nursing education that reproduces scientific racism in the form of deficit thinking about the other (Bell 2020). Racism in nursing education cannot be eradicated by a single person or fleeting movement. Every nurse must continue to be subject to it (Burnett et al. 2020). The nursing curricula should, among others, be based on social justice principles that seek to care for the community in a communal sense.

Nursing education needs to be transformed significantly in order to fit the African setting. As one looks for relevant education that will benefit everyone and reduce the equity gap between citizens, the reasons and objectives of decolonised education must be clarified and redefined. In neglected areas, such as rural areas, nurses should take on the role of health educators by bringing in pertinent programmes that will aid in society’s fight against illness. Nursing education should work to de-gender and de-racialise the nursing profession (Msila 2017).

The identified antecedents were awareness and identification of colonial knowledge and thought, hegemonies, colonial attitudes, colonialism, racism, exclusion and denial colonial systems and content in curriculum and colonial legacies on nursing education systems. According to Kerr (2014), nurse educators need to be aware and conscious of colonial relations and practices that affirm the perpetuation of the domination of Western epistemic perspectives in academic spaces. It is important that nursing education should decentre the idea of the white supremacy epistemology that other systems and orientations of knowing are uncivilised, irrational, and superstitious. Furthermore, Kerr (2014) maintains that the complex world of system embodiment in the European capitalist, military, and Christian epistemology needs to be actively sought to be deconstructed. In order to promote ontological multiplicity in the knowledge-making practice of critical work in nursing education, nurse educators need a critical coloniality lens that draws on critical theory and critical pedagogy. This lens should ask nurse educators to explore their own geopolitical relation to colonial privilege. The decolonisation process requires educators to shift the fundamentally complicated classroom environment that is focused on whiteness, the privileged social position associated with European ancestry, and whiteness.

According to Ali et al. (2021) decolonisation of the curriculum refers to the creation of spaces and resources for a dialogue among all members of the universities on how to imagine and envision all cultures and knowledge systems in the curriculum, and with respect to what is being taught and how it frames the world. Such discourse requires deconstruction, rethinking and reconstruction to make curricula inclusive and representative of different communities, voices, and perspectives.

### Process of decolonisation of the curriculum

A decolonised curriculum must put South Africa and Africa at the centre of teaching, learning, and research and put these perspectives, knowledge, and thinking on an equal footing with the dominant Western canon, in addition to incorporating the epistemic perspectives, knowledge, and thinking from the African continent and the global South. The colonialists’ existing hegemonic histories, ideologies, and methodology must be revisited, unlearned, and rewritten to fully decolonise a society. In addition, it advocates for embracing bodies and traditions of knowledge and knowledge-making in innovative and experimental ways from all other sources.

The absence of curriculum transformation is another let-down. Black faculty and students have been permitted access to ‘white’ areas since 1994, but they are nevertheless expected to follow the rules and refrain from challenging or upsetting the status quo. The curriculum is intertwined with the institutional culture and, given that the latter remains white and Eurocentric at the historically white institutions, the institutional environment is not conducive to curriculum reform, which has had a profoundly negative impact on curriculum transformation. Most university courses still have a European focus, are grounded in knowledge systems from the colonial and apartheid era and are disengaged from the reality and experiences of black South Africans (Arday et al. 2021).

English (2021) is of the view that settler nurse researchers and educators must start the process of decolonising nursing education. The settler must yield, create space, and make sure that indigenous voices are prominent in curricula to start decolonising nursing education. It is the responsibility of the nursing educator to balance knowledge and wisdom and to think about how to establish and maintain the environments that make this possible. There is a need to evaluate who continues to profit from colonisation. Furthermore, nurse educators must reflect on their practical experience and realise that they too must consider the long-term effects of colonisation on indigenous communities and people. A common foundation of nursing knowledge is evidence-based or evidence-informed practice, but whose evidence is this? Does it reflect on whose knowledge and realities are being used in education? English (2021) further posits that defending the value of evidence-based approaches without questioning what knowledge is relevant and reflected in the available evidence is problematic. By creating a global health curriculum, learning objectives, and competences, the nursing curriculum can be decolonised.

### Consequence of decolonisation

Africa must continue to be at the centre of South African epistemology, knowledge, instruction, learning, and research. The curriculum will not just become localised, isolated, or Africanised as a result of decolonisation. Universities still have a duty to turn out graduates who can survive in a complex, interconnected society. Through decolonised curricula, students would be made aware of South Africa’s position and the concerns that surround it on the African continent and in the larger world. Indigenous knowledge systems, culture, and methods of knowing ought to be included in the curricula. Decolonised curriculum must be relevant, appropriate, and meaningful for local, national, and continental settings in order to be used in the complex, interconnected, and unfair world.

The curriculum needs to find the correct balance between past and present injustices, structural dominance, oppression, and exploitation in South Africa, African continent, and the rest of the world, as well as the abilities and information required to overcome these in the future (McGregor & Park 2019; Zembylas 2017). Heleta (2016) is of the view that by incorporating the epistemic perspectives, knowledge, and thinking from the African continent and the global South, the decolonised curriculum will put South Africa and Africa at the forefront of teaching, learning, and research. It will put them on an equal footing with the nursing curriculum’s predominately Eurocentric focus. Decolonisation of the curriculum should be approached from the bottom up by progressive academics and students rather than waiting for institutions and administrators to figure out and approve a ‘decolonisation framework’, come up with an implementation plan, and then possibly even implement it at some point in the distant future. With the aid of the students, progressive academics can decolonise their own curriculum (Rowe et al. 2015).

### Theoretical definition

Decolonisation of the curriculum refers to deconstruction, rethinking and fundamental transformation of colonial knowledge, thought, content and colonial legacies in nursing education curriculum and systems to Africanise knowledge, create new forms of knowledge that include different indigenous knowledge systems using paradigms of indigenous knowledge and worldviews that informs self-determination practices, teachings, and experiences.

### Theoretical validity

The report’s capacity to clearly define and explain a notion is known as its theoretical validity (Hayashi et al. 2019). The meaning of decolonisation of the curriculum in nursing education was identified after the researcher reviewed 52 pertinent primary and secondary materials on the definitions, nature, characteristics, and uses of decolonisation. Decolonisation was given a theoretical definition that illustrated its distinctive characteristics (the epistemological principle). A model scenario was created to demonstrate the application, significance, and use of the features of decolonisation, and concept analysis clearly established the result and empirical referents (the pragmatic principle).

### Implications for nursing education and practice

The implication is that nurse educators should consciously and intentionally seek to design curricula that evidence decolonisation of nursing epistemology and knowledge generation. Indigenous knowledge systems and practices must be the fundamentals of nursing curricula in South Africa. Local communities need to participate in the development of nursing curricula, and nursing education spaces, knowledge and sources must be decolonised to be relevant and make sense to indigenous communities. Indigenous nursing scholars should undertake focused research that seeks to discover and unearth indigenous knowledge and practices that will enable nurse practitioners to embrace indigenous knowledge in order to be efficient, effective, and relevant in their practice (Kimani 2023). Nursing practice needs to acknowledge, confront, and transform oppressive practices in the nursing profession. All nurse practitioners should actively take responsibility to ensure that nursing practice is culturally congruent and culturally responsive to the beliefs of the individuals, families, groups, organisations, and communities that they serve. Policy developers in practice must scrutinise taken-for-granted assumptions that perpetuate oppression and racism (Smith 2019). Practice must focus on decolonised evidence-based practice that embraces indigenous healthcare knowledge and practices.

## Conclusion

The time is now to concentrate on purposely and intentionally decolonising the nursing curriculum in South Africa to care for the various communities. Nurse educators are urged to concentrate on challenging the preconceived notions about how nursing should be performed, as decided by the profession’s alleged founders. Instead of viewing nursing education through a single lens, they should start embracing indigenous knowledge and ways of life that will help African communities in the nation achieve self-determination and work to address the social injustices that Western science and medical procedures have contributed towards. One needs to stop acting in ways that aim to devalue and disparage indigenous knowledge as primitive and unsophisticated.
